# Supplementary material for: Pan-cancer proteogenomic interrogation of the Ubiquitin Proteasome System
Source: bioRxiv. 2026 May 10:2026.03.23.713741. Originally published 2026 Mar 26. Preprint. [Version 2] doi: 10.64898/2026.03.23.713741 (PMC13041857; doi:10.64898/2026.03.23.713741)
Supplement: Supplement 5 [file NIHPP2026.03.23.713741v2-supplement-5.pdf]

bioRxiv preprint doi: https://doi.org/10.64898/2026.03.23.713741; this version posted May 10, 2026. The copyright holder for this preprint (which was not certified by peer review) is the author/funder, who has granted bioRxiv a license to display the preprint in perpetuity. It is made available under aCC-BY 4.0 International license.

786 **Supplementary Figure Legends**

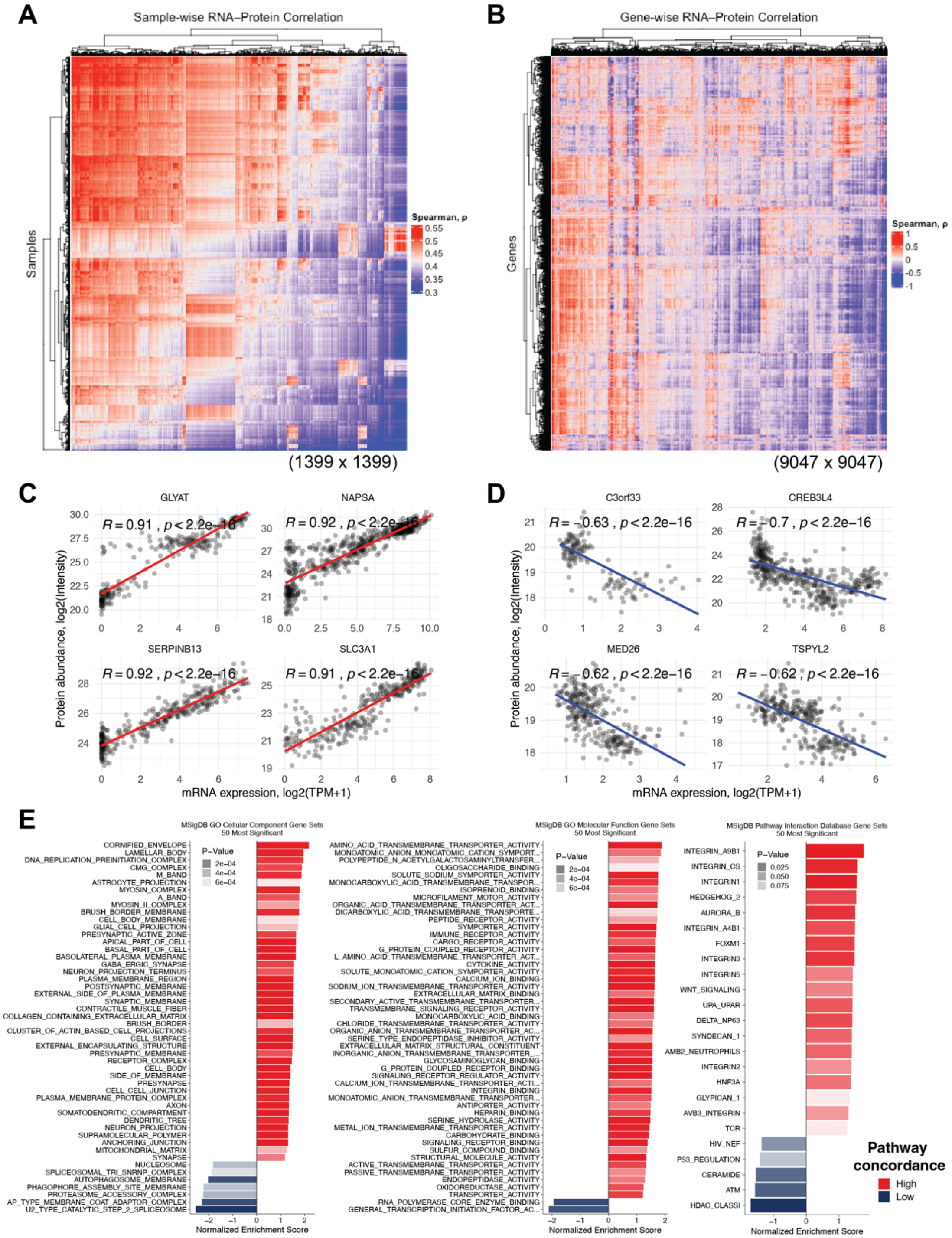


787

788 **Supplementary Figure 1. Sample- and gene-level mRNA-protein concordance in CPTAC.**

789 (A) Heatmap of sample-wise Spearman correlations between matched mRNA and protein

790 profiles across CPTAC cohorts. (B) Heatmap of gene-wise Spearman correlations between

bioRxiv preprint doi: https://doi.org/10.64898/2026.03.23.713741; this version posted May 10, 2026. The copyright holder for this preprint (which was not certified by peer review) is the author/funder, who has granted bioRxiv a license to display the preprint in perpetuity. It is made available under aCC-BY 4.0 International license.

mRNA and protein abundance. (C) Representative scatter plot of a gene with positive mRNA–protein correlation. (D) Representative scatter plot of a gene with negative mRNA–protein correlation. (E) Bar plots of pathway-level mRNA–protein concordance.

bioRxiv preprint doi: https://doi.org/10.64898/2026.03.23.713741; this version posted May 10, 2026. The copyright holder for this preprint (which was not certified by peer review) is the author/funder, who has granted bioRxiv a license to display the preprint in perpetuity. It is made available under aCC-BY 4.0 International license.

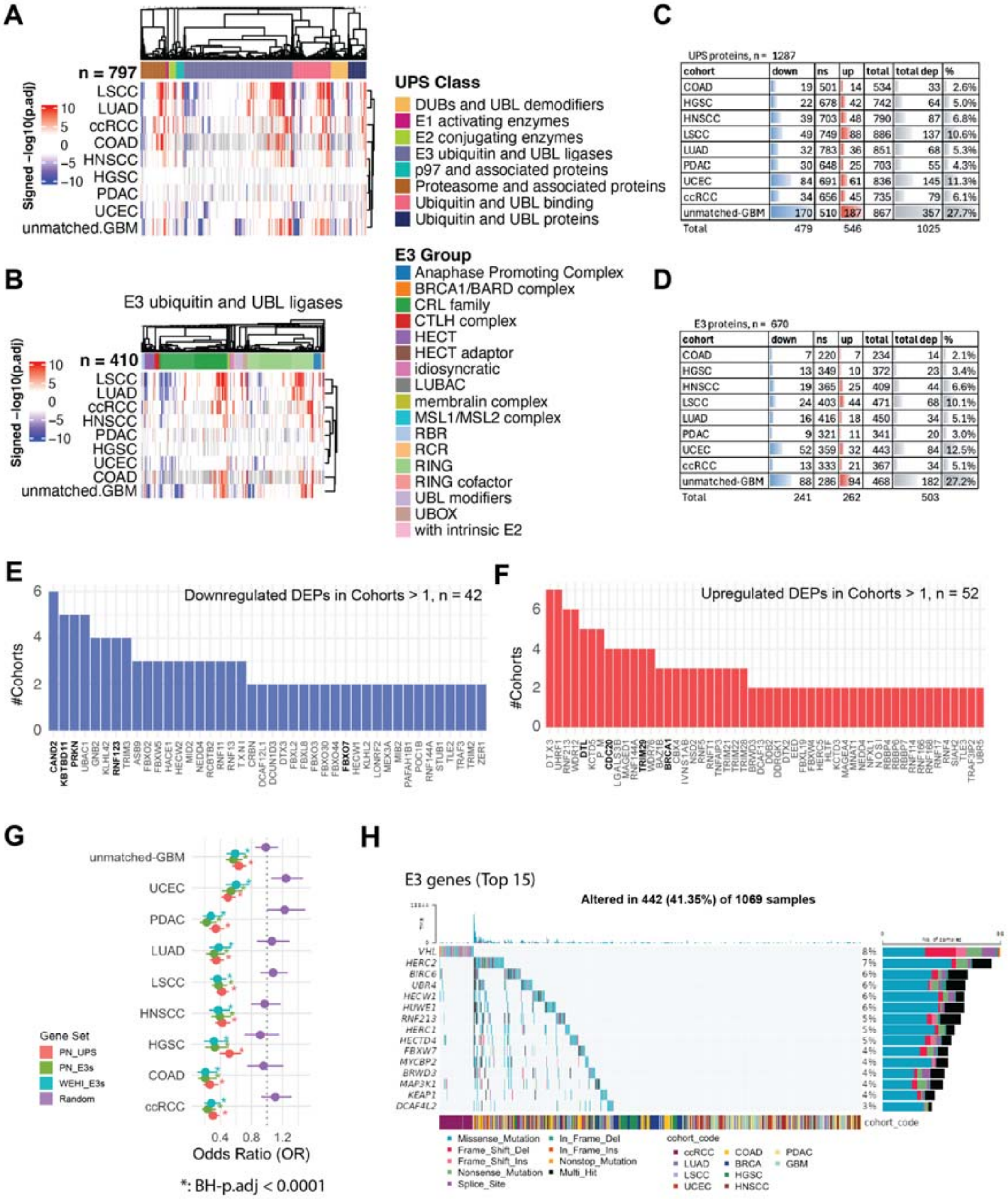


**Supplementary Figure 2. Differential expression landscape of UPS and E3 proteins.** (A) Heatmap of signed –$\log_{10}$(adj. *P*) values for UPS-associated proteins (tumor vs. normal) across CPTAC cohorts. (B) Heatmap of signed –$\log_{10}$(adj. *P*) values for E3 ligases across cohorts. (C) Table summarizing the number/proportion of UPS-associated proteins significantly up- or downregulated in each cohort. (D) Same as (C) for E3 ligases. (E) Bar plot of significantly

bioRxiv preprint doi: https://doi.org/10.64898/2026.03.23.713741; this version posted May 10, 2026. The copyright holder for this preprint (which was not certified by peer review) is the author/funder, who has granted bioRxiv a license to display the preprint in perpetuity. It is made available under aCC-BY 4.0 International license.

downregulated UPS proteins across cohorts. (F) Bar plot of significantly upregulated UPS proteins. (G) Fisher's exact tests comparing dysregulation frequency among UPS-associated proteins versus randomly sampled protein sets. (H) Oncoplot of frequently mutated E3 ligases across CPTAC cohorts.

bioRxiv preprint doi: https://doi.org/10.64898/2026.03.23.713741; this version posted May 10, 2026. The copyright holder for this preprint (which was not certified by peer review) is the author/funder, who has granted bioRxiv a license to display the preprint in perpetuity. It is made available under aCC-BY 4.0 International license.

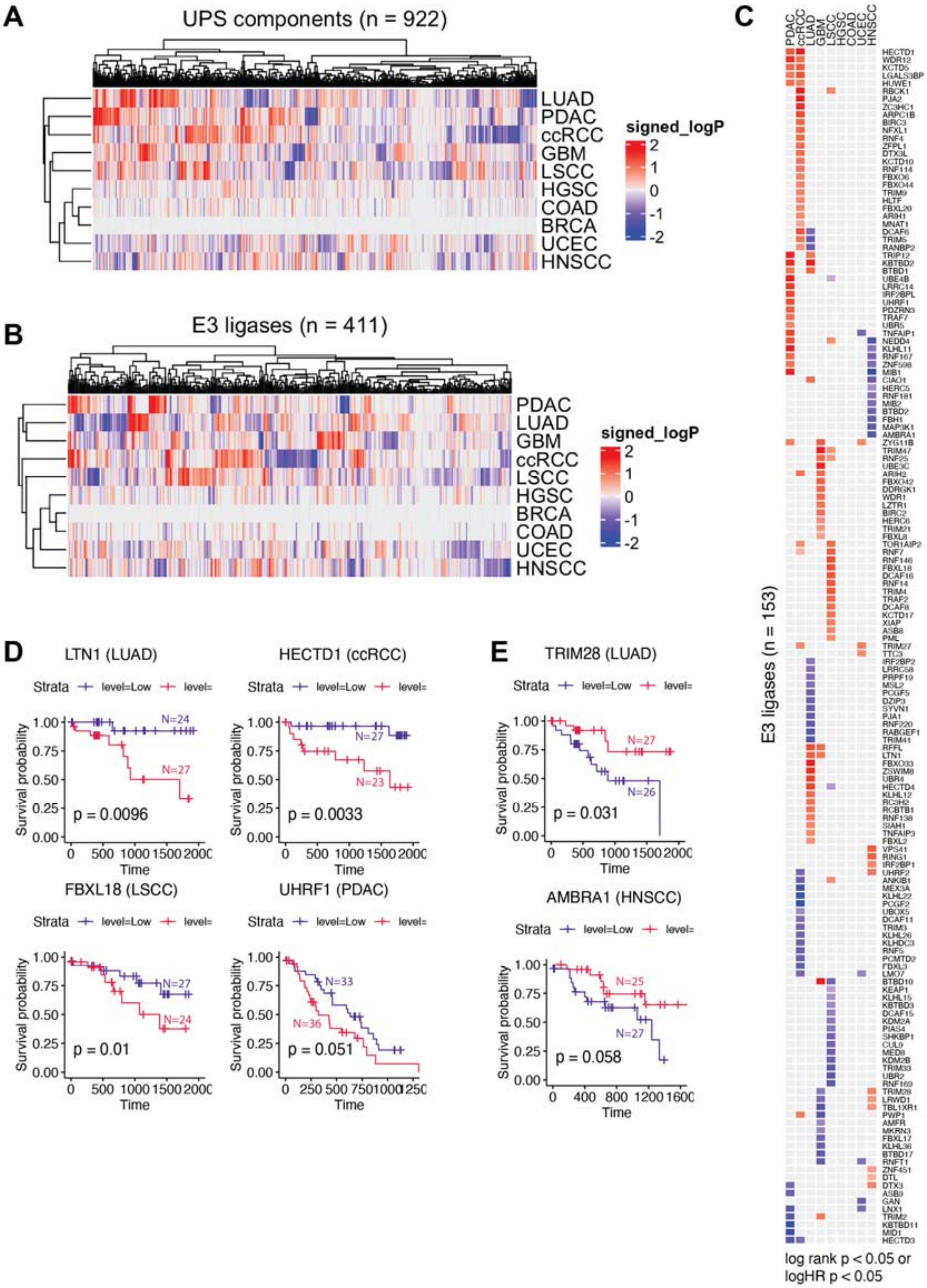


**Supplementary Figure 3. Survival associations of UPS components and E3 ligases.** (A) Heatmap of pan-cancer overall-survival associations for UPS-associated proteins (Cox log-

bioRxiv preprint doi: https://doi.org/10.64898/2026.03.23.713741; this version posted May 10, 2026. The copyright holder for this preprint (which was not certified by peer review) is the author/funder, who has granted bioRxiv a license to display the preprint in perpetuity. It is made available under aCC-BY 4.0 International license.

hazard ratio). (B) Same as (A) for E3 ligases. (C) Set of E3 ligases (n = 153) with significant overall-survival associations in at least one tumor type. (D) Representative Kaplan–Meier curve for an E3 with poorer survival when high. (E) Representative KM curves for E3s with improved survival when high.

bioRxiv preprint doi: https://doi.org/10.64898/2026.03.23.713741; this version posted May 10, 2026. The copyright holder for this preprint (which was not certified by peer review) is the author/funder, who has granted bioRxiv a license to display the preprint in perpetuity. It is made available under aCC-BY 4.0 International license.

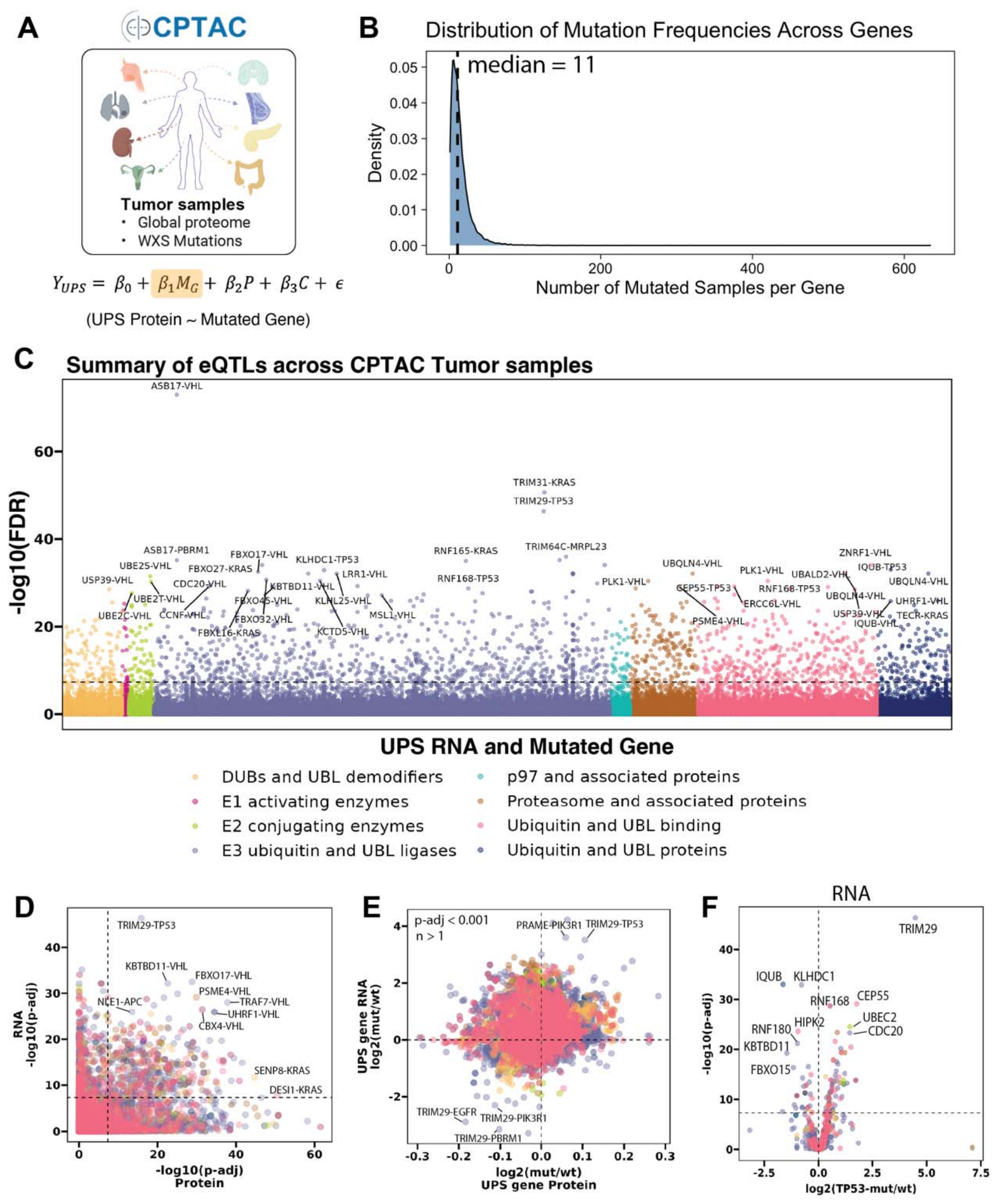


**Supplementary Figure 4. Expanded mutation-to-expression quantitative-trait-locus analysis.** (A) Schematic of CPTAC datasets and the linear-modeling framework (Eq. 1). (B) Density plot of mutant-sample counts per recurrently mutated gene across CPTAC cohorts. (C) Manhattan plot of transcript-level mutation-to-expression QTL associations. (D) Scatter plot comparing $-\log_{10}$(adj. $P$) values from mRNA- vs. protein-level mutation associations for UPS

bioRxiv preprint doi: https://doi.org/10.64898/2026.03.23.713741; this version posted May 10, 2026. The copyright holder for this preprint (which was not certified by peer review) is the author/funder, who has granted bioRxiv a license to display the preprint in perpetuity. It is made available under aCC-BY 4.0 International license.

genes. (E) Scatter plot comparing $\log_2$ fold changes from mRNA- vs. protein-level mutation associations. (F) Volcano plot of *TP53*-mutation effects on UPS-associated mRNA abundance.

bioRxiv preprint doi: https://doi.org/10.64898/2026.03.23.713741; this version posted May 10, 2026. The copyright holder for this preprint (which was not certified by peer review) is the author/funder, who has granted bioRxiv a license to display the preprint in perpetuity. It is made available under aCC-BY 4.0 International license.

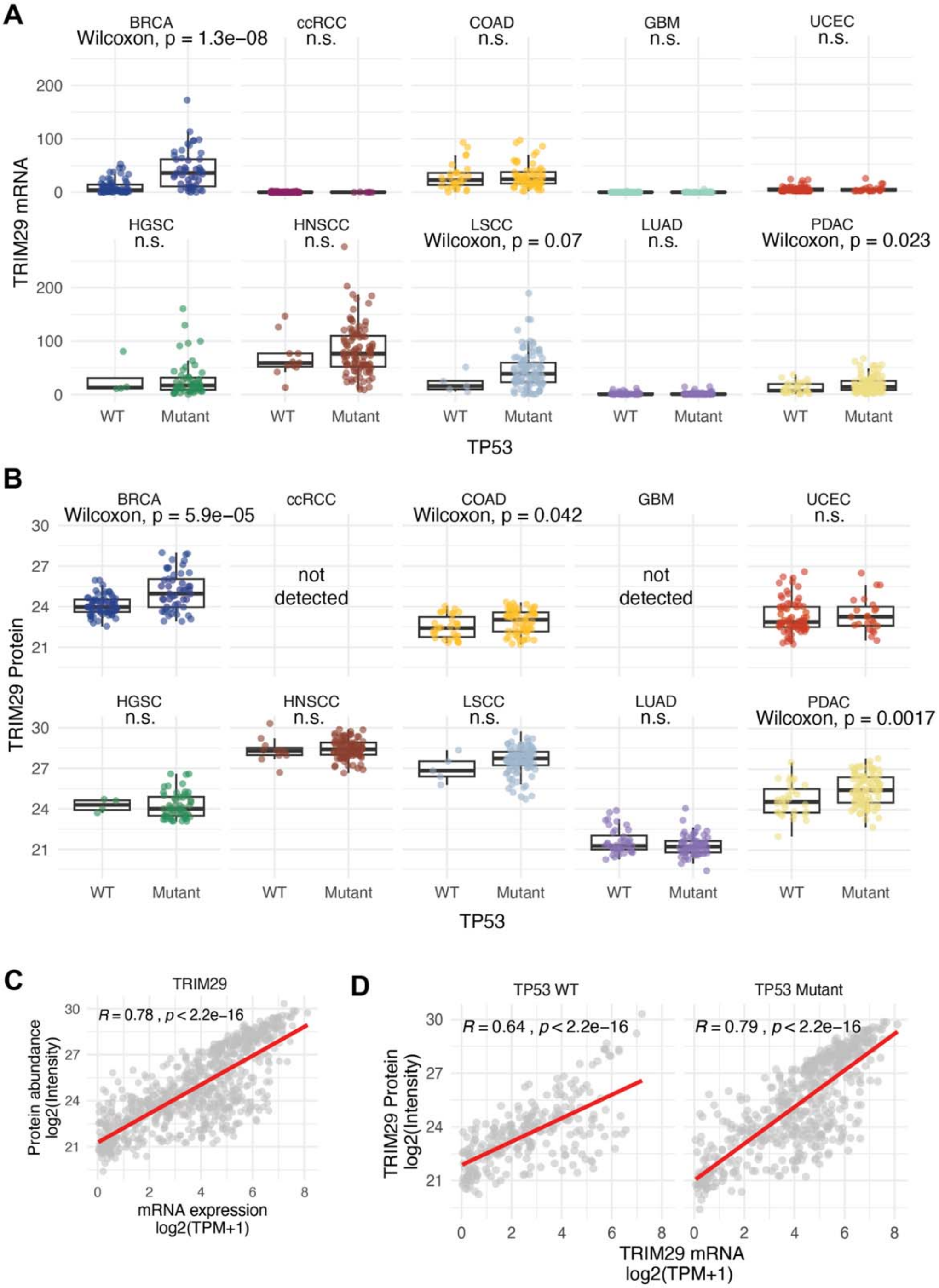


**Supplementary Figure 5. TRIM29 expression stratified by *TP53* mutation status.** (A) Distribution of TRIM29 mRNA abundance in *TP53*-mutant vs wild-type CPTAC tumors. (B) Distribution of TRIM29 protein abundance, same stratification. (C) Scatter plot of TRIM29 mRNA vs protein abundance across CPTAC tumors. (D) Same as (C), stratified by *TP53* status.

bioRxiv preprint doi: https://doi.org/10.64898/2026.03.23.713741; this version posted May 10, 2026. The copyright holder for this preprint (which was not certified by peer review) is the author/funder, who has granted bioRxiv a license to display the preprint in perpetuity. It is made available under aCC-BY 4.0 International license.

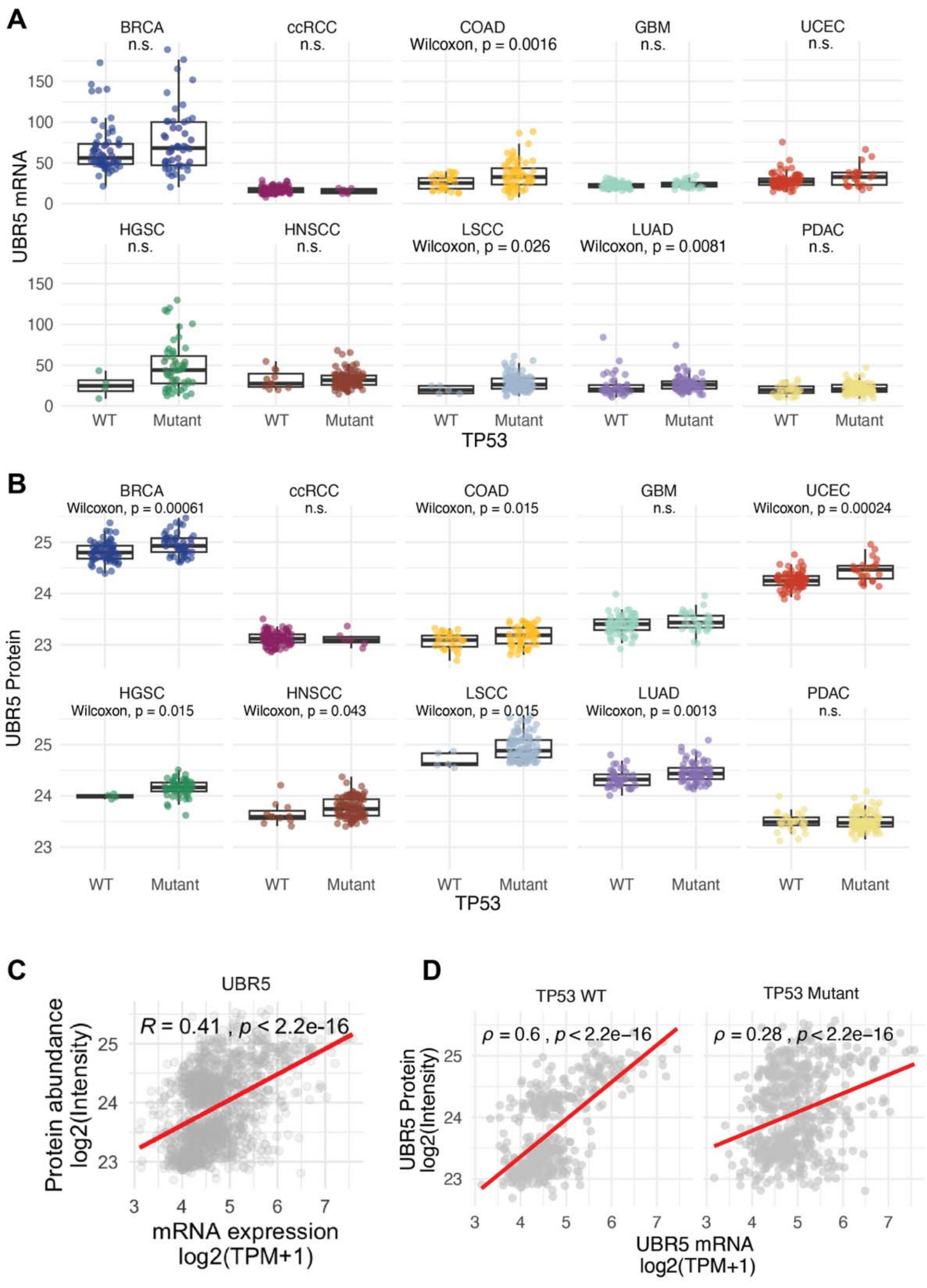


**Supplementary Figure 6. UBR5 expression stratified by *TP53* mutation status.** (A) Distribution of UBR5 mRNA abundance in *TP53*-mutant vs wild-type CPTAC tumors. (B) Distribution of UBR5 protein abundance, same stratification. (C) Scatter plot of UBR5 mRNA vs protein abundance across CPTAC tumors. (D) Same as (C), stratified by *TP53* status.

bioRxiv preprint doi: https://doi.org/10.64898/2026.03.23.713741; this version posted May 10, 2026. The copyright holder for this preprint (which was not certified by peer review) is the author/funder, who has granted bioRxiv a license to display the preprint in perpetuity. It is made available under aCC-BY 4.0 International license.

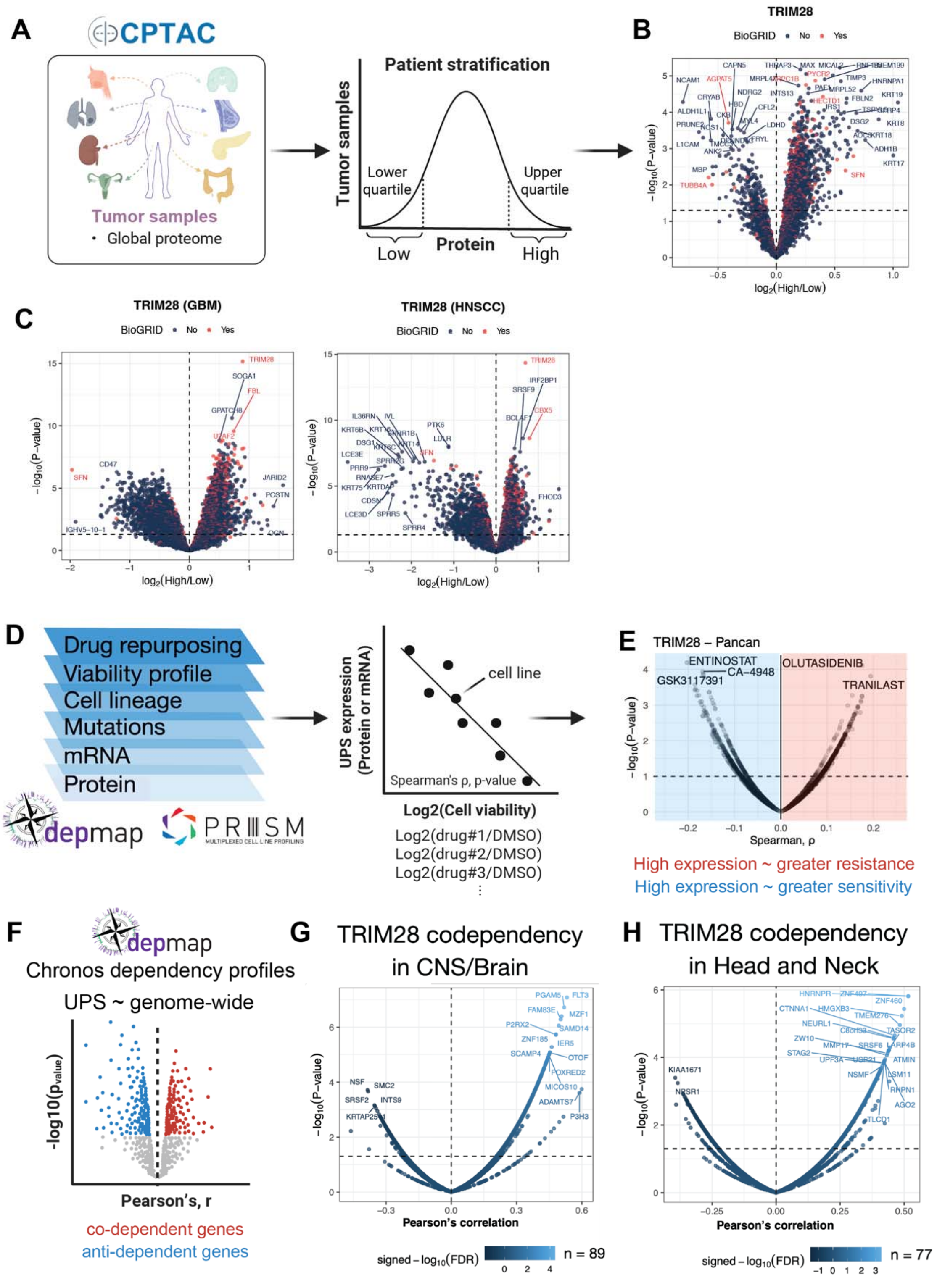


**Supplementary Figure 7. Differential expression, drug sensitivity, and dependency analyses for TRIM28.** (A) Schematic of the differential analysis comparing TRIM28-high and

bioRxiv preprint doi: https://doi.org/10.64898/2026.03.23.713741; this version posted May 10, 2026. The copyright holder for this preprint (which was not certified by peer review) is the author/funder, who has granted bioRxiv a license to display the preprint in perpetuity. It is made available under aCC-BY 4.0 International license.

TRIM28-low tumors across CPTAC cohorts. (B) Pan-cancer volcano plot of differential protein abundance (TRIM28-high vs low). (C) Lineage-specific volcano plots (GBM, HNSCC). (D) Schematic of the drug-sensitivity analysis. (E) Pan-cancer volcano plot of TRIM28–PRISM associations. (F) Schematic of the co-dependency analysis. (G) Volcano plot of TRIM28 co-dependency in CNS/brain. (H) Volcano plot of TRIM28 co-dependency in HNSCC.

bioRxiv preprint doi: https://doi.org/10.64898/2026.03.23.713741; this version posted May 10, 2026. The copyright holder for this preprint (which was not certified by peer review) is the author/funder, who has granted bioRxiv a license to display the preprint in perpetuity. It is made available under aCC-BY 4.0 International license.

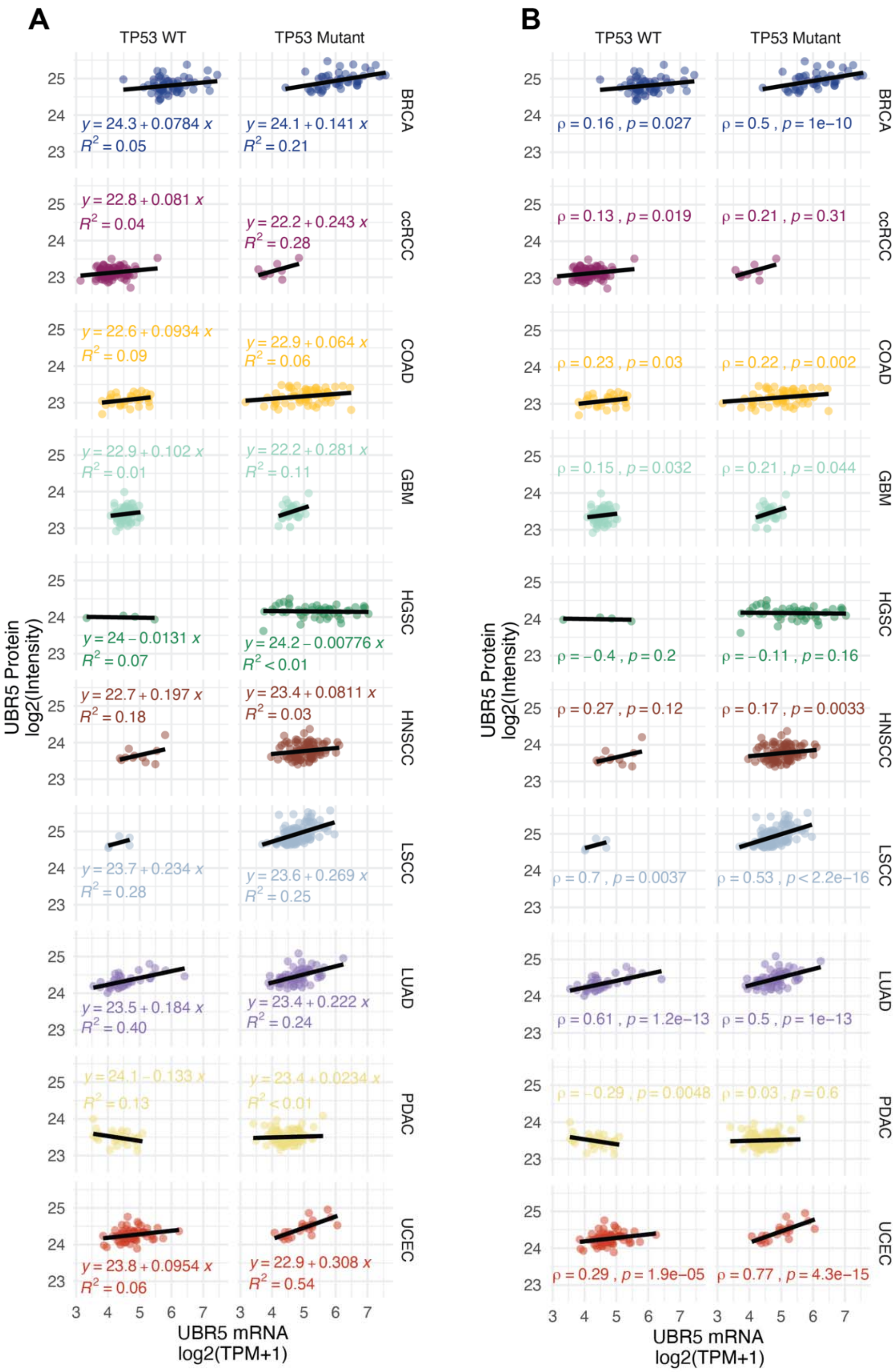


**Supplementary Figure 8. RNA–protein concordance for UBR5.** (A) Linear regression of UBR5 mRNA vs protein abundance across CPTAC tumors. (B) Gene-wise Spearman correlation between UBR5 mRNA and protein abundance.

bioRxiv preprint doi: https://doi.org/10.64898/2026.03.23.713741; this version posted May 10, 2026. The copyright holder for this preprint (which was not certified by peer review) is the author/funder, who has granted bioRxiv a license to display the preprint in perpetuity. It is made available under aCC-BY 4.0 International license.

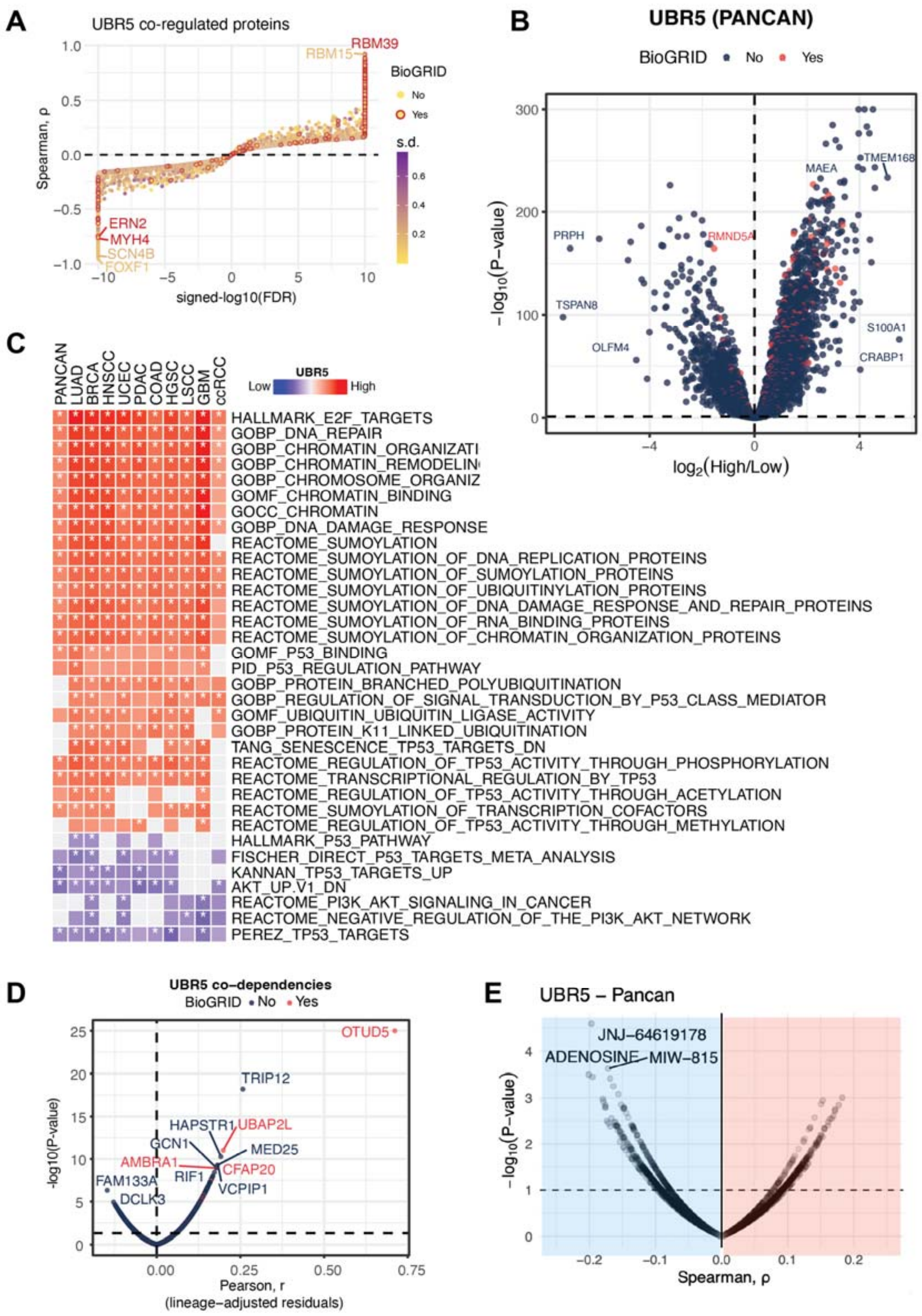


**Supplementary Figure 9. Pan-cancer UBR5 protein co-regulation and functional associations.** (A) Pan-cancer UBR5 protein co-regulation profile (Spearman's correlation). (B)

bioRxiv preprint doi: https://doi.org/10.64898/2026.03.23.713741; this version posted May 10, 2026. The copyright holder for this preprint (which was not certified by peer review) is the author/funder, who has granted bioRxiv a license to display the preprint in perpetuity. It is made available under aCC-BY 4.0 International license.

Volcano plot of pan-cancer UBR5 protein co-regulation. (C) Heatmap of GSEA results from UBR5 protein co-regulation at pan-cancer and lineage-specific resolution. (D) UBR5 co-dependency profile from DepMap CRISPR Chronos with BioGRID interaction annotations. (E) Volcano plot of UBR5–PRISM drug sensitivity.

bioRxiv preprint doi: https://doi.org/10.64898/2026.03.23.713741; this version posted May 10, 2026. The copyright holder for this preprint (which was not certified by peer review) is the author/funder, who has granted bioRxiv a license to display the preprint in perpetuity. It is made available under aCC-BY 4.0 International license.

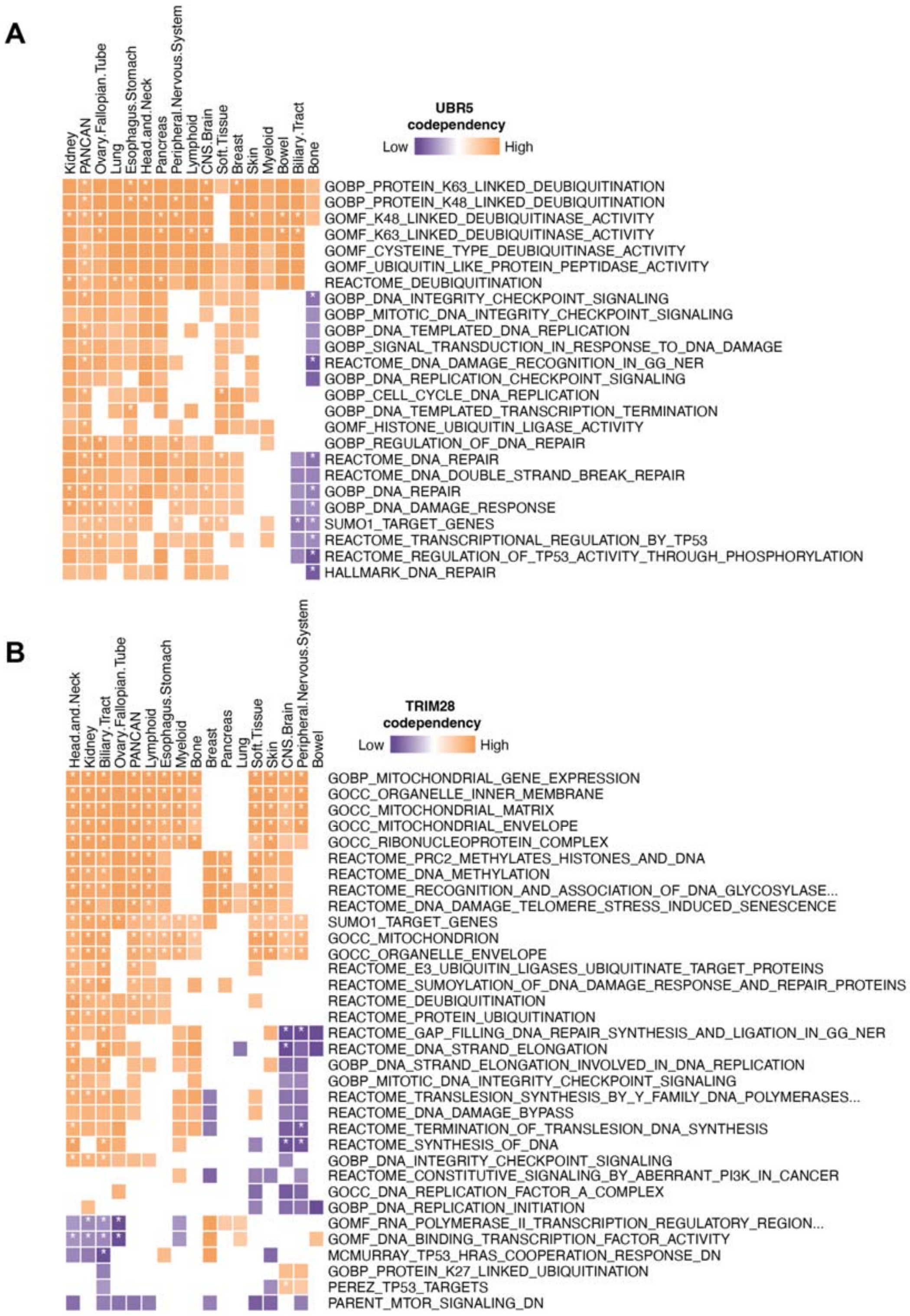


**Supplementary Figure 10. Pathway enrichment based on co-dependency profiles.** (A) GSEA heatmap derived from UBR5 co-dependency profiles at pan-cancer and lineage-specific

bioRxiv preprint doi: https://doi.org/10.64898/2026.03.23.713741; this version posted May 10, 2026. The copyright holder for this preprint (which was not certified by peer review) is the author/funder, who has granted bioRxiv a license to display the preprint in perpetuity. It is made available under aCC-BY 4.0 International license.

resolution. (B) GSEA heatmap derived from TRIM28 co-dependency profiles at pan-cancer and lineage-specific resolution.

**Supplementary Tables**

- **Supplementary Table 1.** Underlying data for specific main and supplementary figures, including mRNA-protein correlation and pQTL analysis.
- **Supplementary Table 2.** Differential UPS protein expression across CPTAC cohorts (per-cohort tumor vs. normal contrasts; adj. *P*, $|\log_2 FC|$).
- **Supplementary Table 3.** UBR5 and TRIM28 co-regulation, GSEA, co-dependency, and PRISM drug-sensitivity results.
